# Cell damage produced by magnetic fluid hyperthermia on microglial BV2 cells

**DOI:** 10.1038/s41598-017-09059-7

**Published:** 2017-08-17

**Authors:** M. Pilar Calatayud, Elisa Soler, Teobaldo E. Torres, Enrique Campos-Gonzalez, Concepción Junquera, M. Ricardo Ibarra, Gerardo F. Goya

**Affiliations:** 1 Nanoscience Institute of Aragón, University of Zaragoza, Zaragoza, Spain; 2 Department of Condensed Matter Physics, University of Zaragoza, Zaragoza, Spain; 3 Laboratory of Advanced Microscopies (LMA), Zaragoza, Spain; 4 Institute for Health Research Aragon IIS. Department of Biology, Faculty of Medicine, Zaragoza, Spain; 5 Facultad de Química, Materiales-Energía, Universidad Autónoma de Querétaro, C.P. 76010 Qro, Mexico

## Abstract

We present evidence on the effects of exogenous heating by water bath (WB) and magnetic hyperthermia (MHT) on a glial micro-tumor phantom. To this, magnetic nanoparticles (MNPs) of 30–40 nm were designed to obtain particle sizes for maximum heating efficiency. The specific power absorption (SPA) values (*f* = 560 kHz, H = 23.9 kA/m) for *as prepared* colloids (533–605 W/g) dropped to 98–279 W/g in culture medium. The analysis of the intracellular MNPs distribution showed vesicle-trapped MNPs agglomerates spread along the cytoplasm, as well as large (~0.5–0.9 μm) clusters attached to the cell membrane. Immediately after WB and MHT (T = 46 °C for 30 min) the cell viability was ≈70% and, after 4.5 h, decreased to 20–25%, demonstrating that metabolic processes are involved in cell killing. The analysis of the cell structures after MHT revealed a significant damage of the cell membrane that is correlated to the location of MNPs clusters, while local cell damage were less noticeable after WB without MNPs. In spite of the similar thermal effects of WB and MHT on the cell viability, our results suggest that there is an additional mechanism of cell damage related to the presence of MNPs at the intracellular space.

## Introduction

A large amount of *in vitro* and *in vivo* studies, as well as clinical trials, have provided solid evidence that hyperthermia (HT) can significantly improve the clinical outcome of radiotherapy and chemotherapy for the treatment of specific types of tumors in oncology^1^. The mechanisms operating during clinical hyperthermia therapy are related to the impact of thermal stress at the cellular and molecular levels^2^. These cytotoxic processes start to take place above temperatures of ~43 °C as a result of protein denaturation^3^, and neoplastic cells are more sensitive to this effect than normal cells, particularly for subsequent radio- or chemotherapy protocols^4, 5^. The rationale of hyperthermia (HT) is that cell sensitization induced by high temperatures is enhanced in those hypoxic, acidotic and nutrient-deprived areas of a tumor where chemotherapy and radiotherapy are less effective^6, 7^.

A recent alternative to achieve selective heating of target tissues is known as magnetic hyperthermia (MHT). This method is based on the use of magnetic nanoparticles (MNPs) as nanoheaters that release heat by frequent reversal of magnetization caused by an external alternating magnetic field (AMF) coupled to the magnetic moment of the MNPs. When the MNPs are uploaded into the target cells they can absorb energy from the magnetic field, heating the cells from the inside^8^. Although MHT has been proposed many decades ago, it was only recently that this technique got its clinical grade as a stand-alone therapy for glioblastoma and prostate cancer^9, 10^. A number of MHT protocols using MNPs have received approval for clinical use in glioblastoma patients under the EU regulations^9, 11^ a procedure currently known as Magnetic Fluid Hyperthermia or Thermotherapy. The protocols involves direct injection of MNPs into a tumor and, after retention by embolization, the application of AMF with amplitude H_0_ ≈ 8.0 kA/m and frequency *f* = 100 kHz^11^.

Glioblastoma multiforme (GBM) is a paradigmatic example of deep-seated tumors with limited accessibility through conventional surgery^12^, and is considered the most aggressive human brain tumor. Its specific growth features related to the glial cells of origin (i.e., astrocytes, ependymal cells or choroid plexus cells). GBM usually presents a high degree of microglia and macrophage infiltration during tumor’s development^13^, and this connection between glioma and infiltrating macrophages has been reported to be a central part of glioblastoma motility and invasiveness^14^. Microglial cells constitute the 5–20% of the glial population^15^, but this number can grow up to 50% inside malignant brain tumors like GBM^16, 17^. Indeed, it has been proposed that the presence of this large number of macrophages around the tumor mass (called “tumor associated macrophages”, or TAMs) could play a functional role in the survival of this type of tumor^18^, but it is still unclear whether this is an active anti-tumor defense mechanism or a tumor-supportive one^19^. The ‘natural’ presence of these well-located immune cells close to the target regions could be profited by using microglial cells as active cargo vehicles for delivering cytotoxic drugs to the targeted tumor^20^.

There is consensus about the capacity of microglial cells to internalize nanostructures such as nanoparticles^21, 22^ or carbon nanotubes^23^, a property that has been already used to locate gliomas through magnetic resonance imaging^24, 25^. Based on this ability, autologous glial cells could be used to carry the nanoheaters to the tumor, with the obvious advantage of the selective heating of target cells and minimal invasiveness. Using microglial cells as delivery vectors to carry MNPs into glioblastoma for thermotherapy has not yet been proposed, to the best our knowledge. In this report we investigate the effect of magnetic hyperthermia on murine microglial BV2 cells loaded with magnetic nanoparticles, to assess the potential of these cells as Trojan Horses during the first stages of a glial microtumor. The MNPs were designed for both optimal specific power absorption (SPA) performance by tuning the Fe_3_O_4_ nucleus size, distribution, shape and maximum cell affinity to reach the MNPs concentration required to raise the temperature within small cell pellets.

## Experimental Results

The initial steps of the experiments were done using two samples of magnetic colloids having somewhat different morphologies and magnetic behavior, produced by different polymers added *in situ* during the synthesis route. These two magnetic colloids were composed by i) polyacrylic acid–coated MNPs (hereafter labeled as PAA-MNPs) synthesized using an oxidative hydrolysis method described elsewhere, and ii) lauric acid-coated MNPs (labeled as LA-MNPs) produced by thermal decomposition of Fe(acac)_3_ as previously reported in ref. 26 (see also Methods section). These two types of MNPs were tested in parallel to provide more flexibility for choosing the best nanoheaters based on: a) the largest specific power absorption (SPA), i.e., maximum heating efficiency per unit mass, and b) the largest amount of MNPs uptaken by the cells, since the heating capability depends on the final MNP concentration.

**Figure 1:**
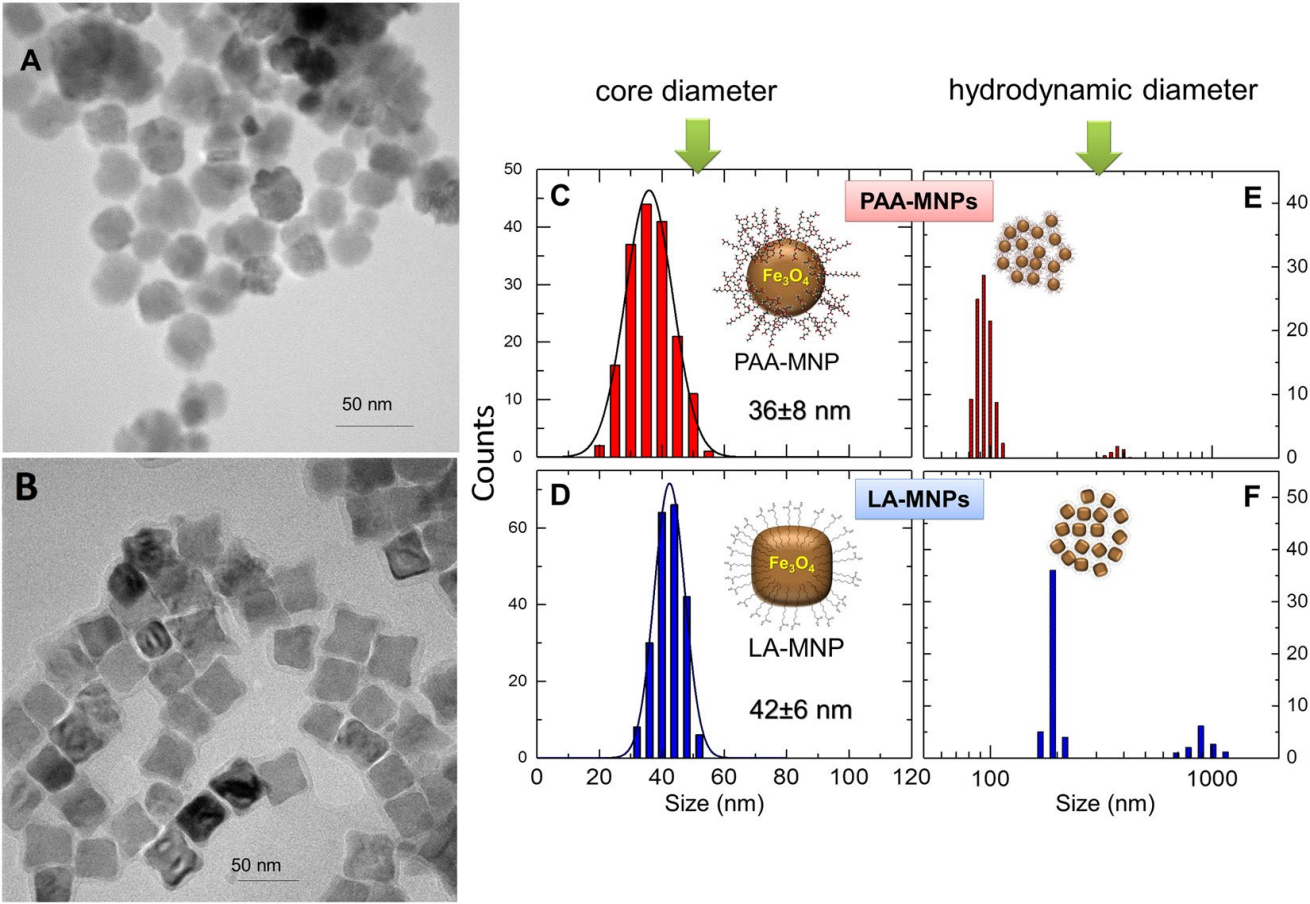
TEM images (left) of PAA-MNPs (**A**) and LA-MNPs (**B**), their histograms (right, **C**,**D**) and hydrodynamic diameter (**E**,**F**).

The morphological analysis of both types of particles through transmission electron microscopy (TEM) analysis showed that the PAA-MNPs have a spherical morphology whereas LA-MNPs have a faceted, octahedral morphology (Fig. 1). The different shapes originate from differences in structural order, and this have an impact on the resulting effective magnetic anisotropy, as will be shown below. The size distribution of the magnetic cores (Fig. 1C and D) from TEM histograms yielded mean values <d> =36 nm (σ = 8) for PAA-MNPs and <d> = 42 nm (σ = 6) for LA-MNPs, whereas the distribution of hydrodynamic diameters as obtained from DLS data (Fig. 1E and F) showed mean values <d_PAA_> = 100 nm (σ = 25 nm) for PAA-MNPs and <d_LA_> = 200 nm (σ = 22 nm) for LA-MNPs. Thus, the *as prepared* aqueous suspensions are mainly constituted of aggregates containing roughly from 8–27 and 64–125 particles for PAA-MNPs and LA-MNPs samples, respectively. A minor but noticeable amount of larger aggregates could also be inferred from the small peak distributions at 400 and 960 nm for both colloids.

As expected from the carboxylic groups of poly(acrylic) and lauric acid, the resulting ξ-potential of both MNPs at physiological pH was negative, −25 ± 3 mV and −17 ± 4 mV for PAA-MNPs and LA-MNPs, respectively. This electrostatic repulsion due to the negative charge provided an effective colloidal stability at neutral pH for several months^27^.

In magnetic hyperthermia, MNPs act as nanoheaters through the energy conversion from the external oscillating magnetic field into heat. The parameter characterizing the heating efficiency of the nanoheaters is the SPA. For the initial characterization of the two *as prepared* ferrofluids, the SPA was measured in triplicate at different frequencies in the range 229 ≤ *f* ≤ 831 kHz and field amplitudes 0 ≤ H_0_ ≤ 23.9 kA/m (Figure S1 of supplementary information). The obtained SPA values represent the power released by the magnetic colloid per unit mass of MNPs. The experimental SPA (W/g) values were calculated from the temperature vs. time data using the expression

1 $$SPA=\frac{{C}_{l}\cdot {\delta }_{l}}{\varphi }\,{[\frac{dT}{dt}]}_{max}$$

where *C*_*l*_ is the specific heat capacity of the solvent, δ_*l*_ the density of the solvent and *ϕ* the mass concentration (kg/l) of MNPs. The ${[\frac{dT}{dt}]}_{max}$ factor in equation (1) was calculated by fitting of the initial slope of the heating curve, (ΔT/Δt)_max_, where ΔT is the temperature increase during the time Δt^28^. The SPA values were measured for both *as prepared* PAA-MNPs and LA-MNPs in water, as a function of the field amplitude H_0_ (at fixed *f*= 560 kHz) and as a function of frequency *f* (at fixed H_0_ = 19.9 kA/m). The resulting data could be fitted with the non-linear expression for specific power dissipation^29^

2 $$SPA({H}_{0},f)=\,{\chi }_{0}\pi {H}_{0}^{2}{\mu }_{0}\,\frac{2\pi {f}^{2}\tau }{1+{(2\pi f\tau )}^{2}}=A{H}_{0}^{2}(\frac{B{f}^{2}}{1+{(Bf)}^{2}})$$

where A is a constant that contains the magnetic properties of the materials, and B is a measure of the relaxation time of the given nanoparticles. Equation 2 is based on the linear response theory (LRT), which assumes that the magnetization M of the system changes linearly with the applied magnetic field according to $M(t)=\,\chi \,H(t)$, where χ is the field-independent magnetic susceptibility. The frequency dependence SPA(*f*) followed Eq. (2) within our experimental range. Regarding the SPA dependence with field amplitude H_0_ (Figure S1), if the LRT approximation is fulfilled the SPA is expected to exhibit a quadratic SPA ∝ H_0_^2^ dependence. To assess deviations from LRT we defined the parameter φ as the power in SPA ∝ H_0_^φ^ so that φ = 2 indicates LRT regime. The fitting of our experimental data within the full range of H_0_ using the relation $SPA\,=\,{\mathbb{A}}\times {H}_{0}^{\phi }$ (with ${\mathbb{A}}={\rm{constant}}$) returned φ values of 3.4 (PAA-MNPs) and 4.4 (LA-MNPs). Indeed, fitting only the data within the low-field region (*H*_0_ ≤ 1  kA/m) yielded even larger φ values, 4.1 for PAA-MNPs and 6.1 for LA-MNPs. The linear response theory (LRT) is expected to be valid for H_0_ < H_k_, where H_0_ is the amplitude of the alternating field and H_k_ = 2K_1_/M_S_ is the anisotropy field of a particle with magnetic anisotropy constant K_1_ and saturation magnetization M_S_^30^. For the present Fe_3_O_4_ magnetite particles M_S_ = 69 Am^2^/kg and assuming the bulk anisotropy constant K_1_ = −11 kJ/m^3^ ^31^ a value of H_k_ ≈ 49 kA/m is obtained, which satisfies the H_0_ < H_k_ requirement, especially for the low-field experiments (H_0_ ≤ 15 kA/m). Therefore the deviations from LRT even at low fields are likely to be originated in the magnetic (dipolar) interactions among MNPs within the agglomerates, an effect that has been recently proven to affect the magnetic energy of the system and thus its heating performance^32–34^.

**Figure 2:**
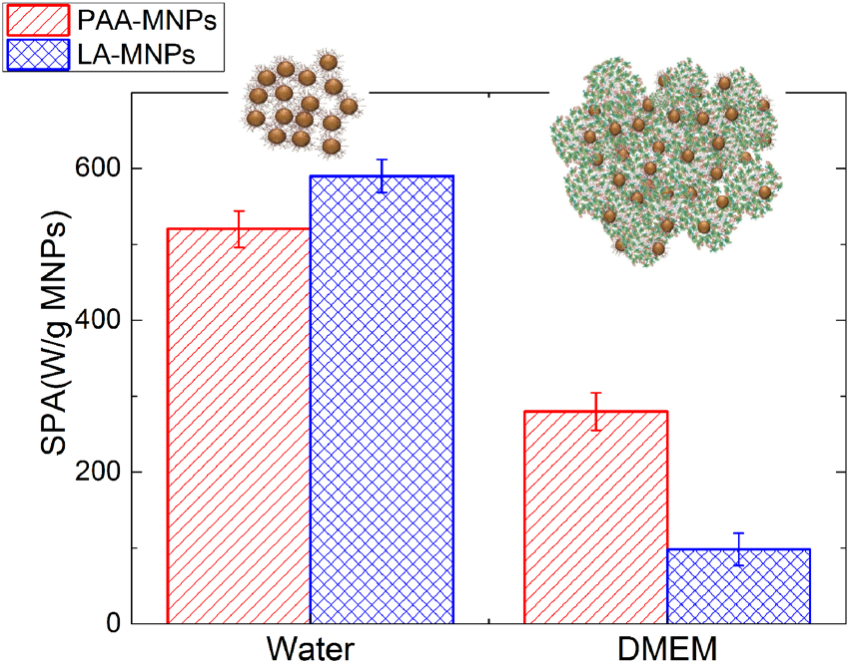
SPA values (H = 23.9 kA/m, *f* = 560 kHz) of both PAA-MNPs and LA-MNPs, as prepared and after dispersing in DMEM cell culture medium.

There is a current general consensus that the viscosity of physiological media can modify the power absorption of MNPs by blocking the Brownian relaxation mechanisms of the magnetic particle^32, 35^. Physical rotation of the MNPs can be blocked either by agglomeration and/or by changes in their hydrodynamic size due to protein absorption, and the magnitude of this blockage on the SPA values depends on the magnetic anisotropy of the MNPs. Thus the power absorption of MNPs in contact with physiological media due to the “protein corona” is a more accurate measurement regarding *in vitro* applications^36^. Accordingly, we determined the SPA value of PAA-MNPs and LA-MNPs in the same biological medium (DMEM) used for the *in vitro* experiments. Figure 2 shows the highest SPA values obtained in water and protein-rich DMEM. The value diminishes notably in both samples and, probably as a combination of protein corona thickness and magnetic dipolar interactions, the relative reduction is more pronounced in LA-MNPs.

### Interactions of Bv2 Cells with Mnps

#### Toxicity and Internalization of Mnps

Assays performed to measure the level of toxicity of both types of MNPs at different concentrations after 24 hours of incubation (Figure S2 of supplementary information) showed nearly 100% viability PAA-MNPs for all concentrations up to 100 μg/ml. For LA-MNPs, the viability results were similar, except for the 100 μg/ml where the viability showed a drop down to 30% of viable cells. Previous works have shown a diminution in cell viability due to lauric acid coatings, although for concentrations lower than in our case^37^.

**Figure 3:**
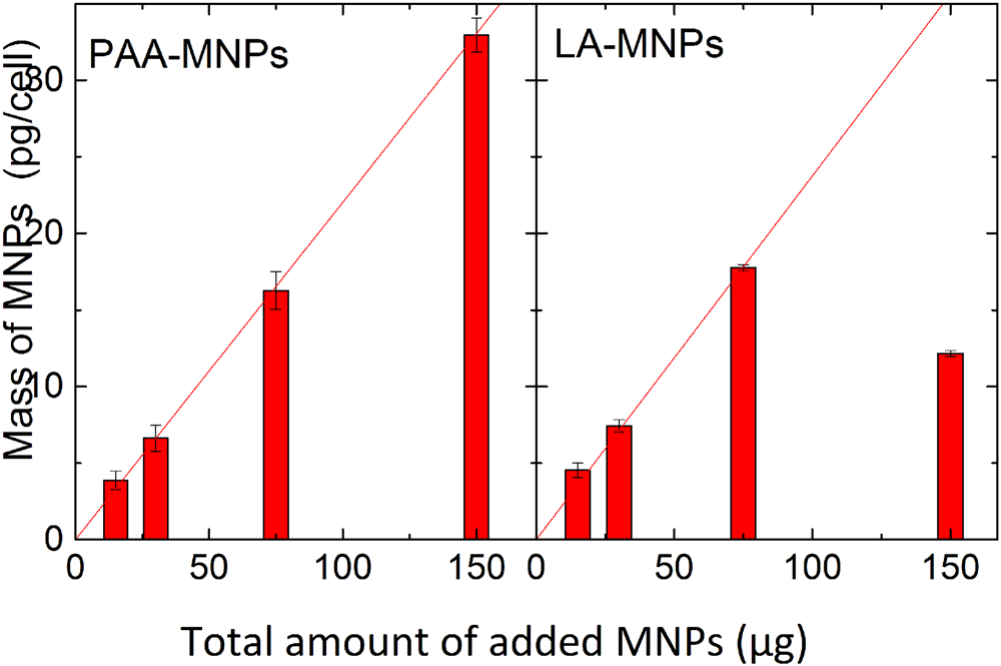
MNPs per cell detected after 24 hours of incubation in BV2 cells as function of MNPs added.

Internalization assays showed efficient uptake of both MNPs by BV2 cells during 24 h. Figure 3 shows a linear trend of MNPs incorporation having similar uptake for both MNPs up to 80 μg added. When cells were cultured with 150 µg of PAA-MNPs and LA-MNPs 35 and 18 pg MNPs/cell cells were found respectively. The low value obtained for LA-MNPs it is related to the toxicity induced at higher concentrations of LA-MNPs. The quantification of MNPs uploaded into the cells as function of total MNPs added displayed a linear trend in the case of PAA-MNPs. A linear fit of this trend gave a slope of 0.22 pg/cell per added μg, i.e., a 22% of the PAA-MNPs added is found in the cells. Similarly, for LA-MNPs the incorporation rate is 0.24 pg/cell*μg added, however at higher concentrations where cytotoxic effect was observed, the incorporation per cell was also affected (see Fig. 3).

Although the efficacy for internalization of both particles was similar, the observed LA-MNPs toxicity at high concentrations led us to choose the PAA-MNPs system as the most adequate material for magnetic hyperthermia experiments. Therefore, the rest of the work described here will refer to the PAA-MNPs as the heating agents for *in vitro* magnetic hyperthermia experiments.

#### Biodistribution of Mnps

**Figure 4:**
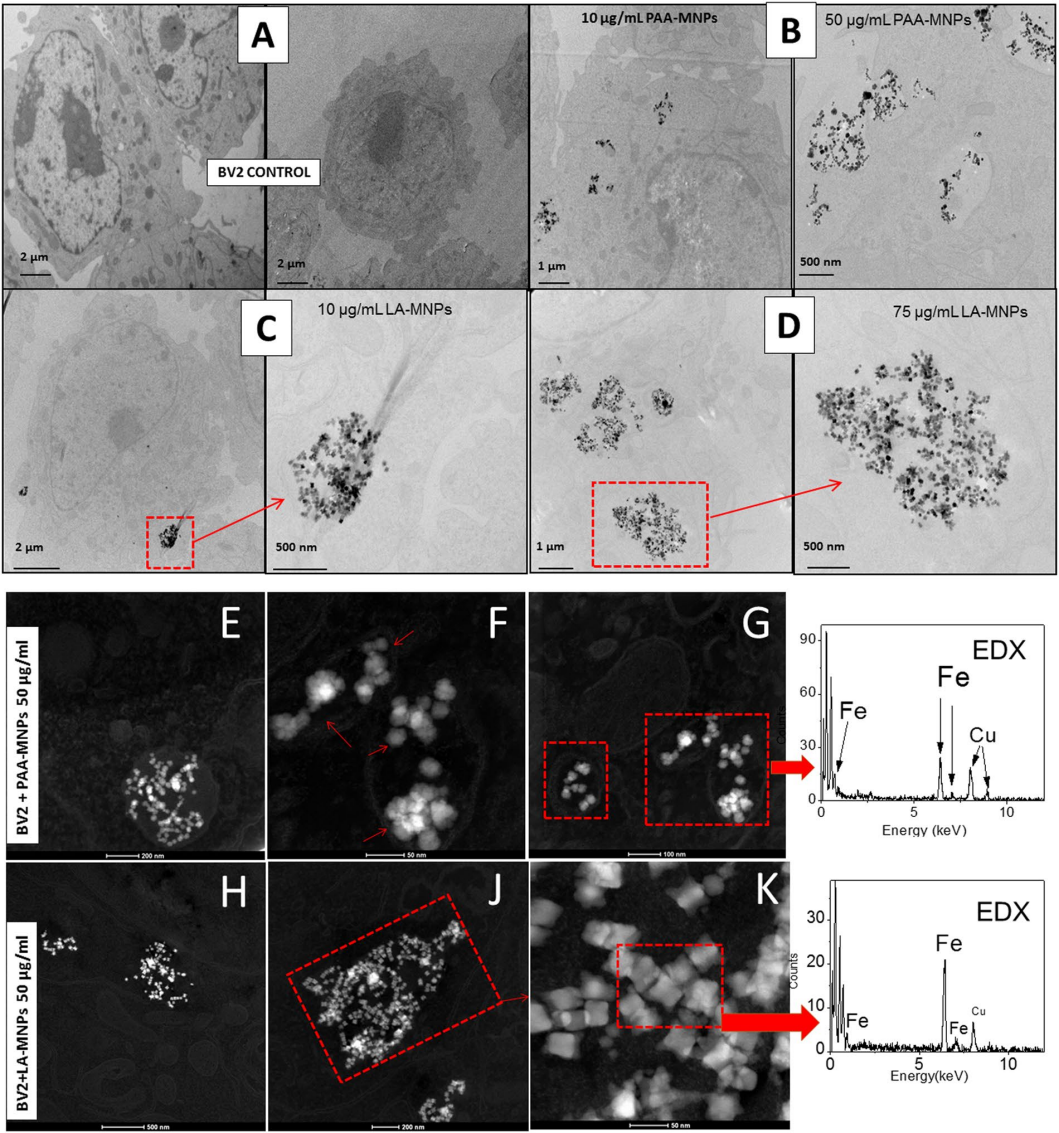
TEM images of BV2 cells with MNPs: (**A**) Control cells (**B**) BV2 incubated with PAA-MNPs 10 and 50 µg/ml; (**C**) BV2 incubated with LA-MNPs at 10 µg/ml and magnified image of the clusters; (**D**) BV2 incubated with LA-MNPs at 75 µg/ml and magnified image of clusters. (**E**–**G**) STEM images of BV2 cells incubated with 50 μg/ml of PAA-MNPs; H-K) STEM images of BV2 cells incubated with 50 μg/ml of LA-MNPs. The last column shows the Energy-dispersive X-ray spectroscopy EDS-HAADF spectra of PAA-MNPs (upper) and LA-MNPs (lower) inside the cell.

The amount of MNPs incorporated by the cells is not the only relevant parameter to be considered for and effective cell death induced by MHT, but also the final location within the intracellular space of those MNPs uptaken^38–40^. The final distribution of MNPs both within the cytoplasm and those attached to the cell membrane were assessed by image analysis using TEM, scanning transmission electron microscopy (STEM) and scanning electron microscopy-focused ion beam (SEM-FIB) dual beam techniques. This information is relevant for the discussion on whether the proximity of MNPs to cell structures could provide additional mechanisms of cell death during magnetic hyperthermia (see below). The intracellular distribution observed by TEM after 12 h of incubation (Fig. 4) confirmed partial internalization of the MNPs, with the presence of particle aggregates surrounded by membranes along the cytoplasm, possibly endosomic vesicles. The preservation of the cell ultrastructure was confirmed irrespective of the concentration of MNPs incorporated by the cells. The STEM-HAADF images and EDS spectra confirmed the Fe fingerprint of the iron-based MNPs inside the cells as brighter spots (Fig. 4), together with the absence of any MNPs inside the cell nuclei.

**Figure 5:**
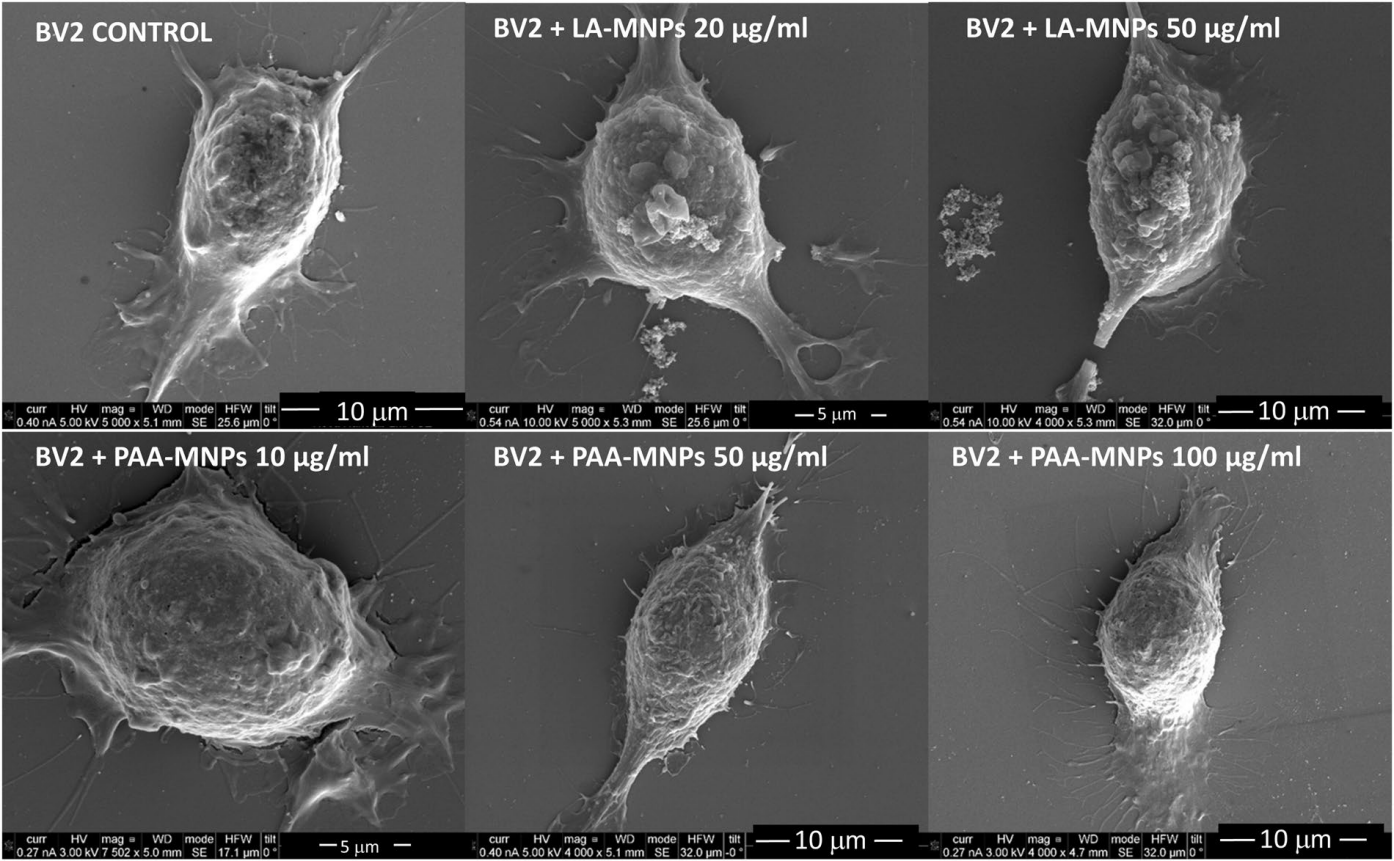
SEM images of BV2 cells incubated at different concentrations of PAA-MNPs and LA-MNPs during 12 h. The short projections from the cell membrane are visible, as well as the major axon-like structures extending from the two opposite ends of the cell poles.

Regarding morphological changes of BV2 cells after incubation with MNPs, SEM images (Fig. 5) showed that the control samples (i.e., cells without MNPs) retained their spherical morphology with flat protrusions and good adherence to the substrate. As a general observation, most of these cells presented many short ‘outcrops’ projecting from the cell body while others displayed two major projections extending from two opposite ends of the BV2 poles. All samples cultures incubated with either PAA-MNPs or LA-MNPs (at any concentration) presented similar morphology. The dimensions, outer surface structure, microglia adherence and general shapes did not appear to be influenced by the treatment with nanoparticles.

**Figure 6:**
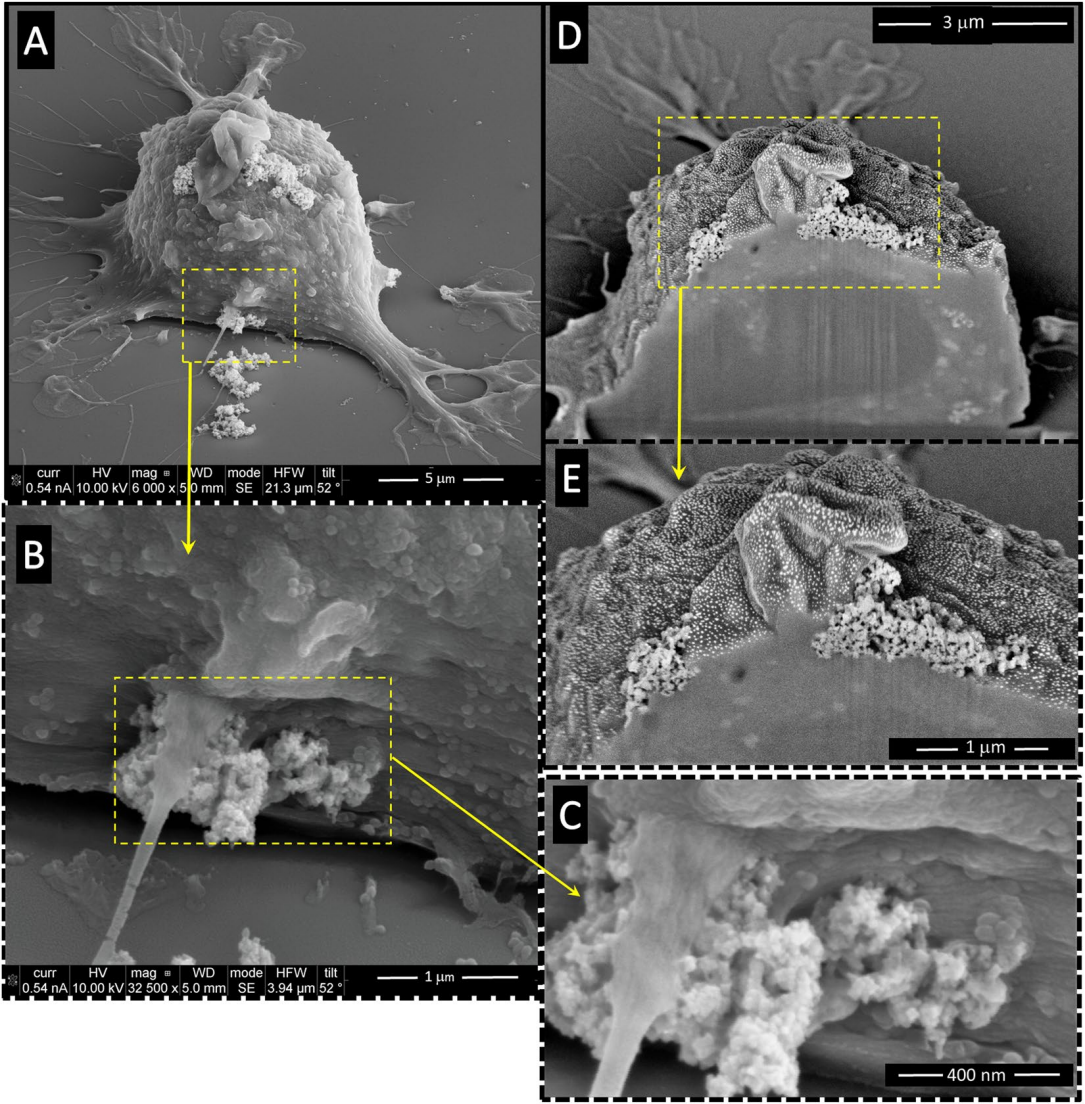
SEM-FIB (Dual–Beam) images of BV2 cells incubated with PAA-MNPs (50 μg/ml, ≈12 pg/cell) showing several clusters pinned at different points of the cell membrane (**A**,**B** and **C**). Cross-sectioning of one cell at one cluster location (**D** and **E**) showed clearly the insertion of the agglomerates through the cell membrane and penetration into the cytoplasm (**C** and **D**).

Regarding the presence of aggregates attached to the cell membrane, it was observed that as the concentration of added MNPs increased, the size and the number of the clusters also increased. Comparatively, the number of such aggregates was lower for PAA-MNPs than for LA-MNPs. Further analysis of the MNPs internal distributions were carried out by FIB-SEM (Dual Beam) microscopy. The cells were cross-sectioned by FIB and analyzed by SEM. Figure 6 shows agglomeration of the MNPs in the cytoplasm.

### Magnetic and water bath Hyperthermia

In order to prove that microglia-delivered nanoparticles could induce significant heating of a micro-tumor we conditioned 9 × 10^6^ cells in 150 μl of culture medium for the *in-vitro* magnetic hyperthermia experiments. This volume was enough to rise the temperature of the cells to the target values (up to 48 °C) after 2–3 min, under our experimental conditions of *f* = 560 kHz and H = 23.9 kA/m. As mentioned before, PAA-MNPs particles were selected due to their higher SPA observed in culture medium when compared to the LA-MNPs values. Achieving the target temperatures is such a short time using AMF reflected the good heating performance of PAA-MNPs regarding a) elevated SPA in biological media (i.e., when mechanical relaxation of the MNPs is blocked), and b) high levels of MNPs internalization by the BV2 cells, rendering the SPA value of the micro-tumor phantom large enough the observed fast heating.

Three control samples were tested: a) blank BV2 cells (i.e., without nanoparticles); b) cells incubated with PAA-MNPs, not exposed to magnetic fields; c) blank BV2 cells exposed to AMF and d) PAA-MNP-loaded BV2 cells exposed to AMF. The first three samples are the controls required to disentangle the effects of MNPs and AMF on the natural viability of BV2 cells. In all cases the viability of the samples was analyzed immediately after the experiments and also after 4.5 h of further incubation. This allows us to assess the direct damage caused to the cells by the AMF treatment and to compare it with those late effects on the cell pathways that could affect viability.

**Figure 7:**
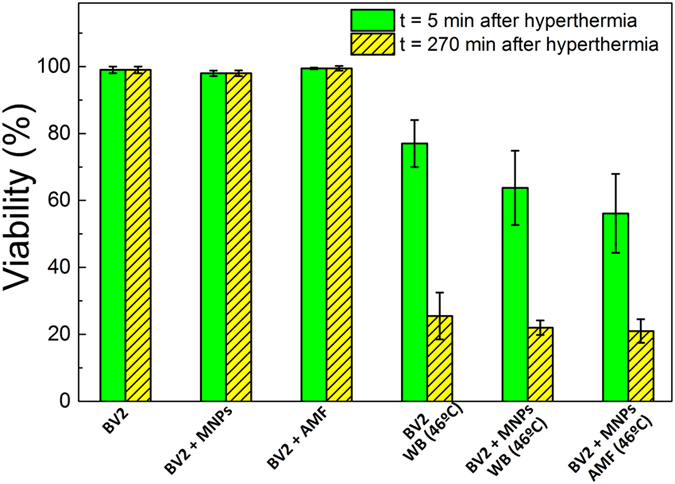
BV2 viability results (n = 3) after water bath and magnetic hyperthermia. Viability was measured 5 min (filled bars) and 4.5 h (dashed bars) after hyperthermia at 46 °C for 30 min. Magnetic hyperthermia experiments were performed at *f* = 560 kHz and H_0_ = 23.9 kA/m.

Figure 7 shows the percentage of cell viability after water bath hyperthermia of BV2 cells and the cells loaded with PAA-MNPs at T = 46 °C during 30 minutes. The viability of three control samples (i.e., samples BV2, BV2+MNPs, and BV2+AMF) remained between 97–99% of the viability of blank cells (BV2), confirming that neither the MNPs nor the AMF, separately, has any measurable effect on the cell viability. On the other hand, the columns in Fig. 7 corresponding to the BV2+MNP+WB, and BV2+MNP+AMF samples with target temperature of 46 °C show a clear drop to 63 and 56%, respectively, after 30 min at the target temperature.

The decrease of the viability up to 50–60% immediately after the application of the AMF increased after 207 min up to ≈25%, irrespective of the heating method, suggesting that a percentage of those cells that remains alive after HT must be metabolically damaged in an irreversible way (see the Discussion section).

**Figure 8:**
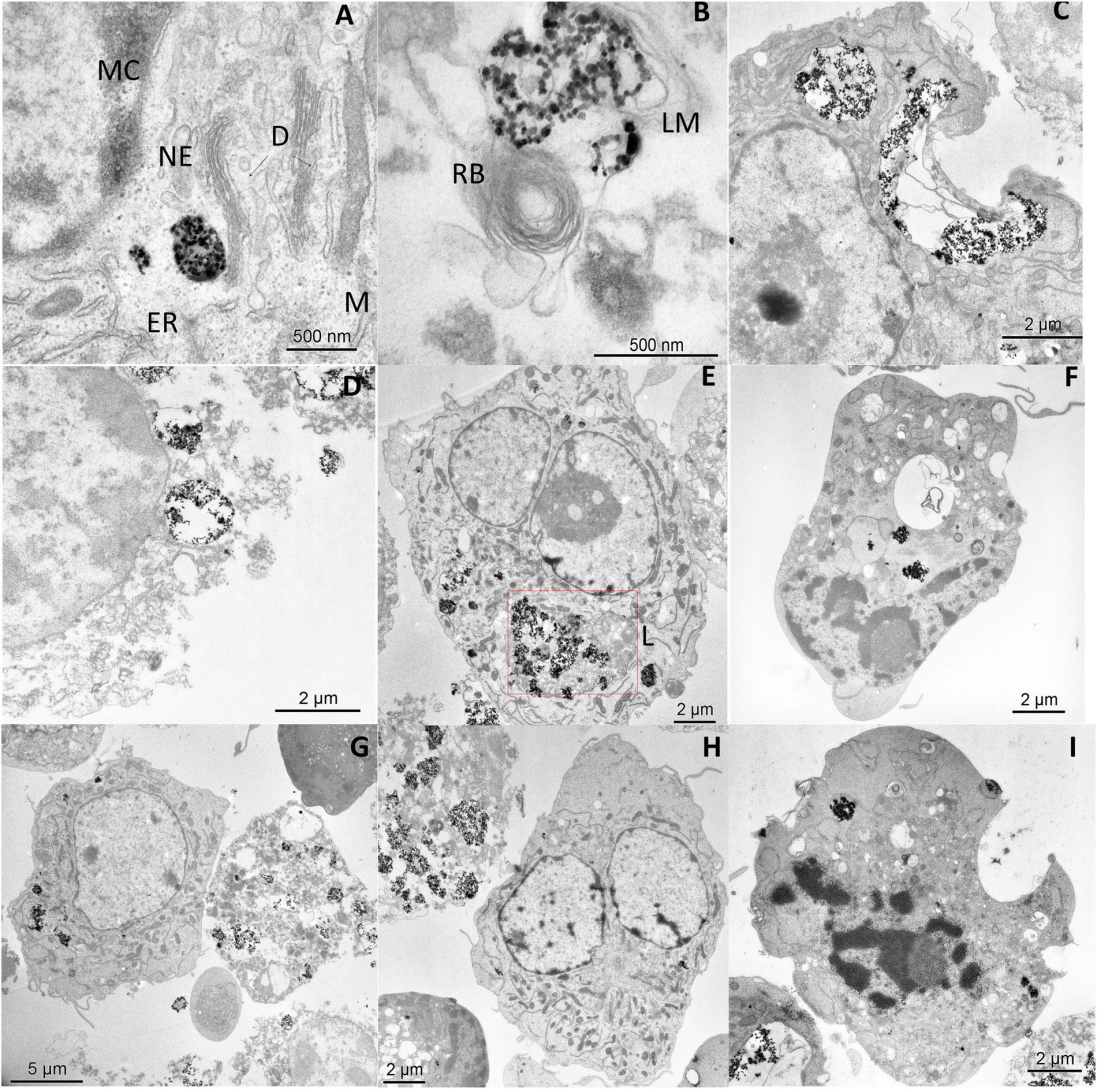
TEM images of BV2 cells containing 100 µg/ml of PAA-MNPs after 30 min of MHT at 46 °C. (**A**) vesicle-containing MNPs, ER: endoplasmatic reticulum, NE: nuclear envelope, MC: marginal chromatine, (**D**) dictyosome and M: mitochondrie. (**B**) MNPs cluster wrapped in lysosomal membrane (LM) and close to a residual body (RB). (**C**) Disrupted cell membrane near a large PAA-MNPs cluster after MHT. (**D**) Typical image of a completely damaged cell due to AMF and PAA-MNPs. (**E**) Cell with lysosome containing PAA-MNPs. Apoptotic cells (**F** and **I**) and dead cells (**G** and **H**) were found to coexist after MHT with (seemingly) alive cells containing high variable amounts of PAA-MNPs incorporated.

TEM analysis of cells submitted to magnetic hyperthermia (Fig. 8) reveals that some of them maintain the internal structural morphology and others are completely injured and show loss of integrity. As observed before, particles tended to aggregate inside the cells causing the break of the cellular membranes (Fig. 8C,D,G and H).

**Figure 9:**
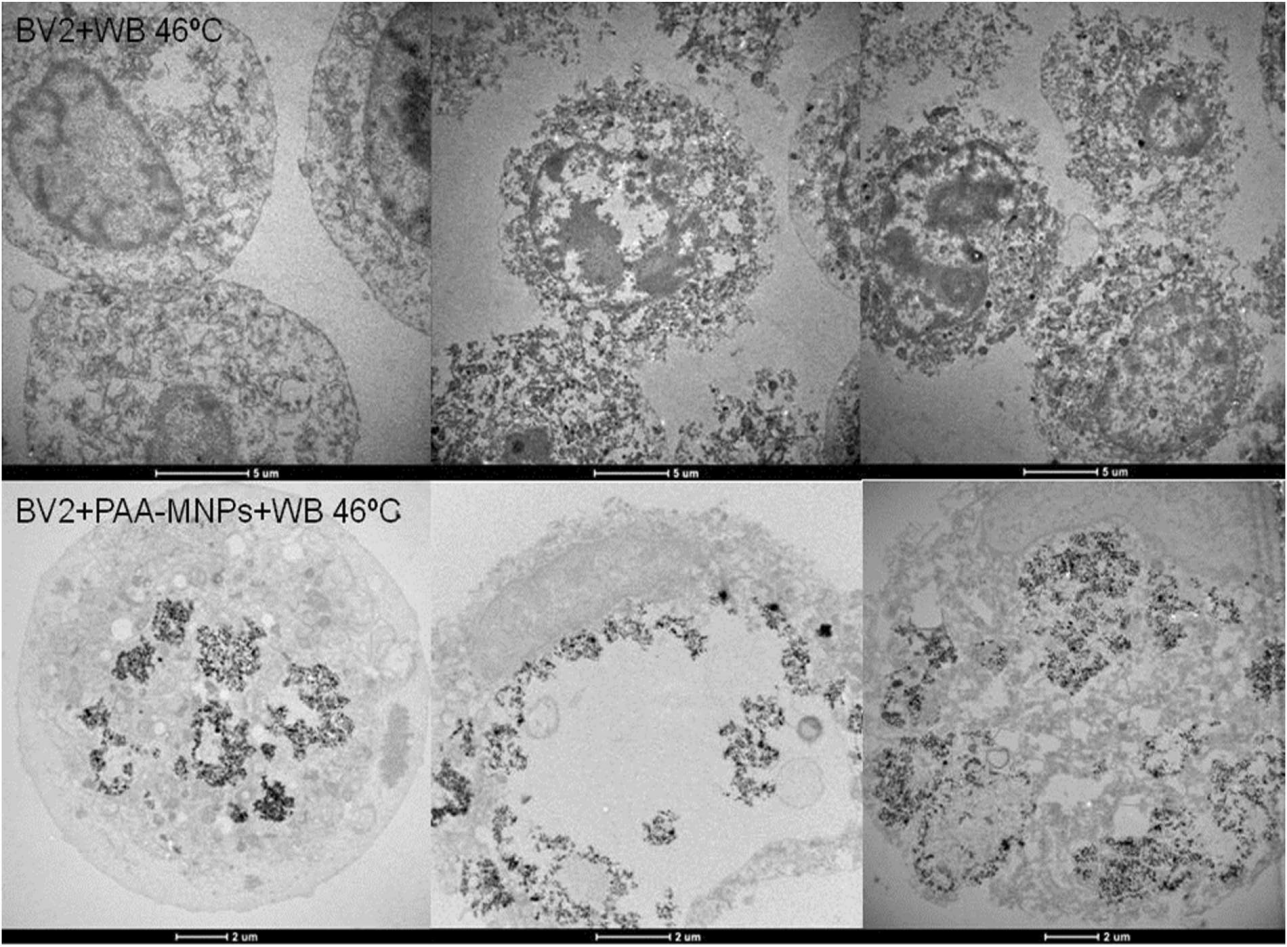
TEM images of BV2 cells after 30 min of water bath hyperthermia at 46 °C. TEM images of BV2 cells containing 100 µg/ml of PAA-MNPs after 30 min of water bath hyperthermia at 46 °C.

Figure 9 shows the TEM images comparing the effects of water bath and MHT protocols on BV2 cells after to 30 min at 46 °C. The most evident feature on all images, common to both protocols, is the loss of structural integrity of the cells, and the presence of vacuoles in the cytoplasm. The cell damage after WB was found to extend across the entire cell population, whereas in the case of MHT a fraction of the cell population loaded with MNPs showed a more pronounced effect of local damage, mainly membrane disruption and changes in organelles. The analysis of these images indicates that the action of the nanoparticles during MHT favors the internal rip of the cells, which would promote a necrotic-like mechanism. No evidence of chromosome condensation was found, although a clear vacuolization process is observed from the images after MHT heating.

**Figure 10:**
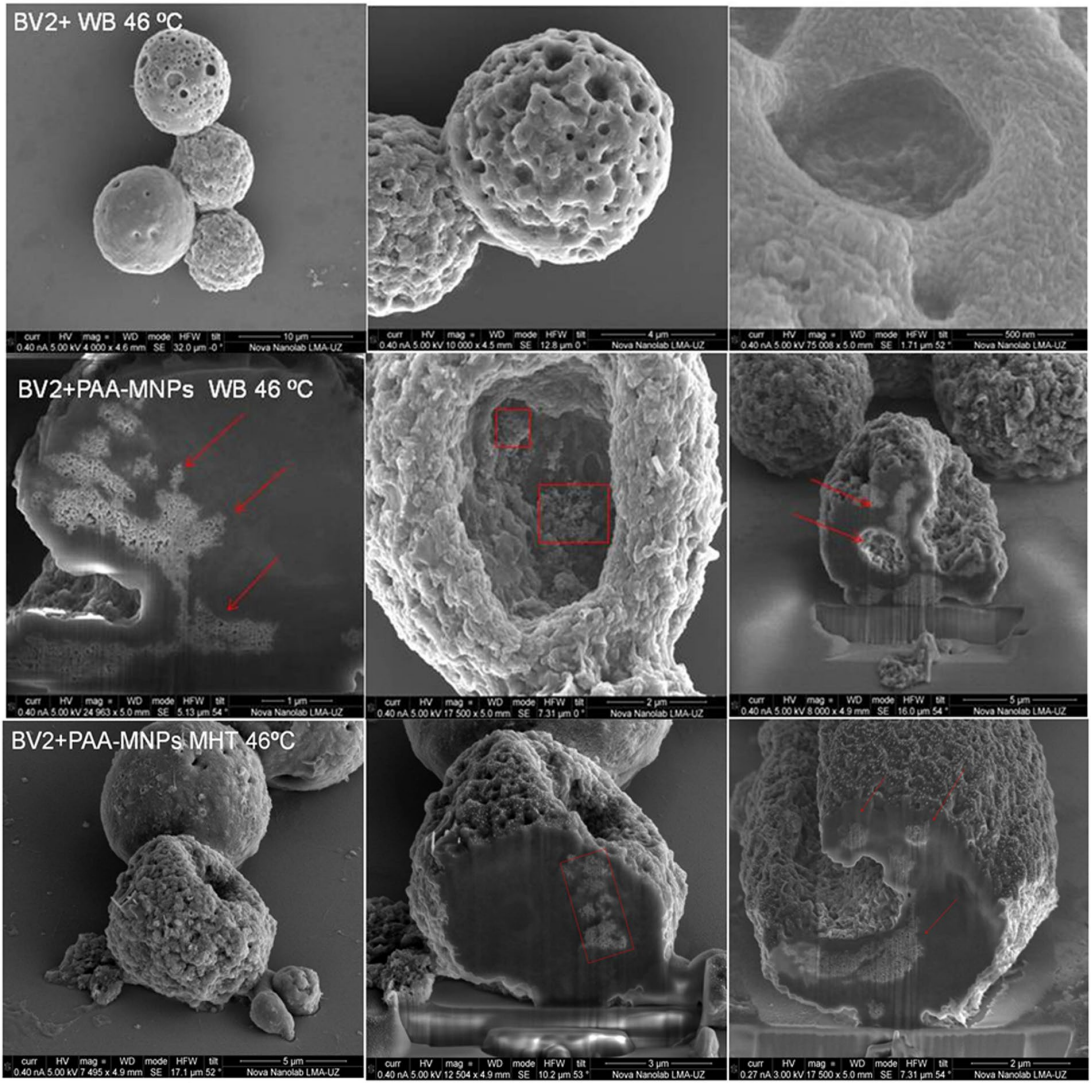
Scanning electron microscopic (SEM) images of BV2 cells after 30 min of water bath hyperthermia at 46 °C. SEM-FIB (Dual-Beam) images of BV2 cells containing 100 μg/ml of PAA-MNPs after 30 min of water bath hyperthermia at 46 °C. Dual Beam images of BV2 + PAA − MNPs + AMF, final Tª = 46 °C; (100 μg/ml PAA-MNPs, 24 k/m, f = 560 kA/m kHz and 30 min).

The analysis by FIB-SEM Dual-Beam of magnetically-loaded cells and cells free of particles after the application of AMF and WB showed clear changes on the external cell morphology (Fig. 10). A completely disrupted cytoplasmic membrane structure is observed, with wide crossing ‘channels’ spread along the surface. The loss of membrane integrity appears to occur at the first stages of the cellular death. The presence of big channels on cell surface explains the membrane permeabilization provoked by the high temperature. From the images of cross-sectioned cells uploaded with nanoparticles we observe that the nanoparticles favor the formation of these channels on cells surface (Fig. 10 middle and lower rows).

## Discussion

The magnetic hyperthermia experiments described above required two main requisites to induce the death of microglial cells: MNPs with good heating efficiency (i.e., large SPA values) and an elevated uptake of MNPs by the cells. Regarding the former, materials engineering is one of the key actions to allow maximizing the SPA and so to minimize the MNPs dose to achieve a therapeutic effect. Many factors influence the heating efficiency (SPA) of magnetic nanoparticles such as particle average size, size distribution, composition, surface functionalization, magnetic anisotropy etc. For magnetite-based MNPs, there is quite an established consensus that enough SPA can be achieved by working with nanoparticle sizes ranging between 25–40 nm^41, 42^. The MNPs used here were designed to have average particle sizes for optimal heating, and on these magnetic cores two different surfactants were tested to provide different MNP-cell interactions. The increase of SPA with increasing *f* of the two *as prepared* colloids could be fitted using equation 2. However, the field dependence was found to follow a power law SPA = A H_0_^φ^, with φ > 2, larger than predicted by the linear response theory (LRT)^29^. As mentioned in the Results section, these deviations are expected because the condition H_0_ << H_K_ is not fulfilled in our MNPs (i.e., the anisotropy fields are H_K_ ≈ 40–50 kA/m), and also because the agglomeration of MNPs results in dipolar interactions that change the energy landscape governing the magnetic relaxation of the system^43, 44^.

The physical mechanisms involved in magnetic relaxation and heat generation are usually approximated by two processes, namely the physical rotation of the particles (Brownian relaxation) and the relaxation of the magnetic moment within the crystal structure (Neel relaxation). In the *as prepared* colloids, it is expected that both Neel and Brown relaxation of the magnetic moment will contribute to the power absorption, yielding SPA values of 600 W/g for LA-MNPs and 520 W/g for PAA-MNPs (*f* = 560 kHz, H = 23.9 kA/m). The decrease of SPA values of both samples in protein-rich culture media (Fig. 2) is explained by the blocking of the Brown (i.e., mechanical) relaxation, originated in the larger hydrodynamic radius and/or agglomeration of the MNPs due to the serum proteins attaching to the MNPs^45^. Although this effect is more pronounced for LA-MNPs (and hence the criterion to select the PAA-MNPs for *in vitro* experiments), our data indicate that Brownian relaxation is a measurable contribution in both La- and PAA-MNPs when they are in water. To keep the relaxation mechanisms unaltered under blocking/agglomeration conditions constitutes the challenge for an optimal design of MNPs for *in vitro* and *in vivo* applications^32^.

The TEM images of Fig. 4 show the presence of MNPs mainly within the cytoplasm space, forming aggregates. The cell ultrastructure is maintained, in contrast with previous results where BV2 cells were seriously damaged after being uploaded with Fe_2_O_3_ NPs^46^. Figure 4A,B and C show the PAA-MNPs wrapped in vesicles with a membrane around the particles, while the LA-MNPs seem to be free in the cytosol (Fig. 4D,E and F). These results are in agreement with other reports that have shown the capability of the BV2 cells to incorporate MNPs and that they were located in cytoplasmic compartments^47^. These cells function as phagocytes of the central nervous system (CNS), which function is to remove cell debris or damaged neurons. They incorporate preferably substances in the range 1–3 μm, and therefore the uptake of MNPs aggregates (50–700 nm) can be due to other non-specific mechanism, electrostatic interactions, van der Waals forces, etc^48^.

The ability of a given cell type to incorporate/attach different amounts of MNPs and the metabolic pathways that determine the final agglomeration state of those particles are important not only for achieving a set point temperature but also to determine the impact of the experiments on cell viability. External parameters such as the AMF values and the number of cells set for each experiment are also to be considered. The success of a hyperthermia treatment will depend on all these factors combined, to produce the expected physical and biological effects. All experiments reported in this work have been aimed to assess the potential of our MNPs to act as efficient cell-killers in a microtumor-mimicking environment^49^.

We remark that the choice of the best MNPs for these hyperthermia studies (i.e. the PAA-MNPs) was determined not by their SPA performance in water, but in cell culture medium under the same conditions (*f* = 560 kHz; H = 23.9 kA/m) and, as a second criterion, the amount of internalized MNPs by the BV2 cell line (up to 35 pg/cell for PAA-MNPs compared to the 17.9 pg/cell of LA-MNPs). Indeed, the concurrent effects of a high SPA value and high cell uptake resulted in a high concentration of MNPs within the compact pellets (9 × 10^6^ cells) and provided a heating rate of about 0.1 °C/s, enough to attain the target temperature (46 °C) within 3–5 minutes. Under these conditions the application to AMF was set to reach the target temperature and keep it constant for 30 min.

The cell viability, as measured five minutes after the hyperthermia protocol described above, dropped to 75% of the control cells. In order to study the long-term effect of AMF a second viability test of the same cells was performed 4.5 h after treatment, resulting in 25% of the control cell viability. Water bath hyperthermia using the same heating time and temperature was also studied on BV2 cells in order to compare with the hyperthermia induced by AMF. No significant differences between both heating methods were apparently detected, having also viabilities of 25% for BV2 and BV2 treated with PAA-MNPs after 4.5 h of water bath. The similar results found in the present work suggest that WB and MFH cause similar structural cell damages. It is known that the changes of macromolecular structures under hyperthermia conditions have a direct impact on the cell cycle through the inhibition of protein synthesis and a breakdown of membranes and cytoskeleton^50^. It is interesting to mention that a systematic study comparing MHT and WB by Ogden *et al*.^51^ using several cytotoxicity assays showed that different thermal doses were necessary to attain similar levels of morphologic and physiologic cell damage. While the direct structural damage on cell membrane or cytoskeleton found by image analysis (Figs 8, 9 and 10) is probably a fast mechanism of cell killing after hyperthermia, some metabolic changes can also go beyond repairing capacity would result in loss of regulation of metabolic key points yielding cell death. Therefore, the differences in cell viability measured at t = 0 and 4,5 h (Fig. 7) shows that metabolic processes are also involved in cell killing by hyperthermia, irrespective of whether it is due to an WB or MHT protocol.

These data are in agreement with other experimental studies evaluating cell death induced in cells pellets after WB and AMF heating^52, 53^. For instance, Wilhelm *et al*. obtained similar cell killing efficiency for human prostatic tumor cells (PC3) treated with maghemite MNPs after 1 h of AMF and 1 h water bath heating in a range of temperatures between 37 to 45 °C. It has to be mentioned that their experimental parameters differed in time of cell exposure, size and volume of cell pellet (cluster of 20 × 10^6^ cells in 300 µl) and AMF conditions^53^. Other reports have found similar percentage of cell survival between magnetic fluid and water bath hyperthermia. They used a larger pellet (5 × 10^7^ − 1 × 10^8^ cells) of human mammary carcinoma line (BT20 cells) uploaded with magnetite MNPs and a magnetic field of 520Khz and 13.2 kA/m during 25 min at 45 °C in^54^. The same group reported similar extent of survival after WB and AMF at 43 and 45 °C at all treatment time tested (20,40,60,90,120 min) for semi-solids pellets of tumor, WiDr human colonic adenocarcinoma cells(1 × 10^8^−1 × 10^9^ cells)^41, 55^. On the other side, some works have reported a better efficiency in particle-induced cell death by magnetic hyperthermia when compared to WB protocols^56^. For example, Sanz *et al*.^57^ have demonstrated that MHT requires an average temperature that is 6 °C lower than that required with a WB protocol to produce a similar cytotoxic effect. Also Rodríguez-Luccioni and co-workers^58^ observed higher effect of AMF on two cancer cell culture models treated with iron oxide NPs (Caco-2: human epithelial colorectal adenocarcinoma: MCF-7:human breast cancer) than WB at 45 °C. The effect was observed 24 hours after 2 hours of application on 3 × 10^6^ cells, with magnetic field amplitude of 20 kA/m and frequency of 238 kHz. It is difficult to establish a comparison among our results and other works reported since the experiments are conducted using different type and number of cells within the pellets, the frequency and amplitude of magnetic field and nanoparticle properties.

It is well known that the heat affects plasma membrane proteins altering the membrane permeabilization and producing cell killing or making the cells more sensitive to additional stress^59^. The FIB-SEM and TEM images of hyperthermia-treated cells showed similar damage induced by the two hyperthermia treatments. The injuries of cellular membrane are clearly observed in those regions where the aggregated nanoparticles are attached, and also the loss of structure along the cytoplasm. In the case of water bath heating (Figs 9 and 10), cellular membranes are broken and the organelles absent in practically all cells analyzed (see more TEM images in supplementary information). Cell membrane structure is specially damaged in the presence of PAA-MNPs whereas BV2 cells free of particles are internally injured but still some cells preserve their membrane. Figure 8 depict the ultrastructure morphology of cells loaded with PAA-MNPs and submitted to AMF. Image A shows a cluster of PAA-MNPs included in a membranous body located close to the nuclear envelope (NE) and marginal chromatin (MC), we can also observe the endoplasmic reticulum (ER), dictyosome (D) of the Golgi apparatus. The PAA-MNPs are close to the Golgi apparatus which will secrete hydrolases that may blend with the endosome containing MNPs and end up forming lysosomes. Also we can observe mitochondria (M) with elongated morphology and perfectly conserved with the double membrane. This cell maintains the structure of all the organelles. Image B displays PAA-MNPs with lysosomal membranes (LM) around and next to a residual body (RB)(vesicles containing indigestible materials).The cells depicted in image C maintain the ultrastructure and the cellular organelles. This image reveals that the vesicles containing the MNPs are peripherally located and they might have suffered a dilation process due to AMF effect, resulting in broken vesicles with membranous residues (marked in the image with an arrow). This process produces the invagination of the plasmatic membrane that forms a round hole comparable to the deformed holes observed in dual-beam images (Fig. 10). The cell exhibited in image D has completely lost the structure of the organelles and the plasmatic membrane that is separated from the cell. The red square of image E marks a lysosome containing PAA-MNPs. Images F and I show two apoptotic cells. Images G and H demonstrate high concentration of MNPs uploaded induces loss of cell integrity and death whereas the cells with fewer amounts of PAA-MNPs incorporated still show the structure of the organelles within the cytoplasm. This means that the quantity of PAA-MNPs incorporated in each cell will influence the level of damage induced when AMF is applied. The force vibration of PAA-MNPs under the effect of an AMF could mechanically destroy the cell membrane structure^60^, produce damages in the interior of the cell, and result in membrane fluidity that triggers cell death^61, 62^. The observed holes in the cell structure were bigger nearby larger clusters of MNPs internalized by the cells, in agreement with previous reports. For instance, Prasad *et al*. reported experiments applying MFH (target temperature T = 43 °C) to HeLa cells loaded with ɣ-Mn_x_Fe_2-x_O_3_ and observed the disruption of the cytoskeleton (actin and microtubules) by immunofluorescence microscopy, suggesting that those changes might be responsible of the observed cell death^63^. Taking into account the lysis of the plasma membrane observed from the Dual Beam and TEM images (see also Figures S3–S5 in Supplementary Information) when the temperature reached 46 °C, it can be supposed that the majority of the cells have died by necrosis mechanism. These results are proof of concept of the efficacy to induce microglial cells death by magnetic and water bath hyperthermia^64, 65^. Moreover, TEM images reveal that cells uploaded with higher amounts of MNPs are more damaged when exposed to AMF compared to cells with small amounts of particles. The mechanism of cell death seemed to induce rapid necrotic cell death originated by the fast increase in the temperature. Despite similar cell survival was obtained for both hyperthermia methods at the target temperature, localized application of magnetic hyperthermia in a tumor might be easier and less aggressive than hyperthermia conventional techniques.

## Conclusions

We have comparatively studied the effect of exogenous (water bath) and intracellular (magnetic hyperthermia) heating protocols on BV2 microglial micro-tumour phantoms. When magnetically loaded with MNPs, we were capable to increase the temperature of small phantoms (≈10^7^ cells, 150 μl volume) up to 48 °C in 2–3 minutes. Regarding the magnetically-loaded cells for MHT, as a general trend we observed that the biodistribution of the MNPs is organized as agglomerates both within the cytoplasm and attached to the cell membrane, with the fraction on cell membrane depending on the MNPs surface properties. Exposure of cells uploaded with PAA-MNPs to target temperatures of 46 °C during 30 min resulted in cell death rates of about 65–75% within 4.5 h after the treatment. No differences were observed in cell viability when the hyperthermia was induced by homogeneous water bath heating or magnetically-triggered. Morphological studies of the PAA-MNPs loaded BV2 cells after magnetic and water bath hyperthermia shows the disruption and channels caused in cells membrane. For *in vivo* application the water bath is a much less specific option, but the results of our publication show that MFH causes the same type of cell damage as the WB heating does. MFH is promising for *in vivo* applications, because it first generates heat and second it delivers MNPs which disrupt the cell structure considerably by forming holes allowing a deep penetration of MNPs and anticancer drugs loaded on them. These results open the possibility for the use of microglial cells as nano-carriers of MNPs to provoke more localized temperature increase in a glioma tumor by magnetic hyperthermia therapy.

## Methods

### Synthesis of Magnetic Nanoparticles (Mnps)

Poly acrylic acid–coated MNPs (hereafter labeled as PAA-MNPs) were synthesized using an oxidative hydrolysis method described elsewhere^27, 36^. Briefly, PAA-MNPs were prepared by mixing a solution containing NaOH and the mild oxidant KNO_3_ with a solution of FeSO_4_ and poly(acrylic acid) polymer (PAA) under N_2_ atmosphere. The resulting mixture was stirred for 24 h at 90 °C. Lauric acid-coated MNPs (LA-MNPs) were produced by thermal decomposition of Fe(acac)_3_ as described previously^26, 66^. The Fe(oleate)_3_ complex used as precursor was synthesized following ref. 67. and then dissolved in a mixed solvent of trioctylamine and benzylether in the presence of oleic acid. The reaction was carried out for 1 h at 330 °C under N_2_ atmosphere. The resulting nanoparticles were transferred to water by forming a bilayer with lauric acid^68^. The Fe_3_O_4_-MNPs concentration in the magnetic colloids was determined by measuring their Fe contents through VIS-UV transmission spectrophotometry (Shimadzu UV-160), based on the thiocyanate complexation reaction^69^:

$${{{\rm{Fe}}}^{3+}}_{(\mathrm{aq})}+{6\,\mathrm{SCN}}_{(\mathrm{aq})}^{-}\,\to {[{\mathrm{Fe}(\mathrm{SCN})}_{{\rm{6}}}]}_{(\mathrm{aq})}^{{\rm{3}}-}$$

PAA-MNPs, LA-MNPs and were dissolved in HCl 6 M-HNO_3_ (65%) at 50–60 °C during 2 h. Potassium thiocyanate was then added to the Fe^3+^ solution to form the iron-thiocyanate complex, which has strong absorbance at 478 nm wavelength. The iron concentration was determined by comparing the sample absorbance to a calibration curve.

### Morphology, Size And Physicochemical Properties of Mnps

MNPs average size, distribution and morphology were analyzed by transmission electron microscopy (TEM) using a FEI Tecnai T20 microscope and operating at 200 keV. High resolution transmission electron microscopic (HR-TEM) images were obtained by using a FEI Tecnai F30 microscope operated at an acceleration voltage of 300 KV. TEM samples of MNPs were prepared by placing one drop of a dilute suspension of magnetite nanoparticles in water on a carbon-coated copper grid and allowing the solvent to evaporate at room temperature. The average particle size <d_TEM_> and distribution width σ were evaluated by measuring the largest internal dimension of more than 200 particles. The zeta potential was evaluated at room temperature on a photo correlation spectrometer (PCS) Brookhaven 90 plus (Zetasizer NanoTM from Malvern Instrument) from a dilute suspension of the sample in water at 0.01 M of KCl. The hydrodynamic diameter distribution (number distribution mode) of the polymer coated nanoparticles in their aqueous suspensions was obtained using a photo correlation spectrometer (PCS) Brookhaven 90 plus (Zetasizer NanoTM from Malvern Instrument). Magnetization measurements were performed by adding 1 mg of sample to 100 μL of ethanol and then immobilizing the particles in epoxy resin. In this way the concentration of all samples were within the ≈1% wt.MNPs range, to minimize dipolar interactions. M vs. H data was taken using a commercial VSM magnetometer (Lake Shore) at 300 K, and magnetic fields −1450 kA/m ≤ H ≤ 1450 kA/m.

### Specific Power Absorption (Spa) Measurements on Colloids

The magnetic hyperthermia experiments were performed using a commercial magnetic applicator device (nB Nanoscale Biomagnetics, Spain). The alternating magnetic field (AMF) could be adjusted to seven different frequencies within the range 229 ≤ *f* ≤ 831 kHz, whereas the field amplitude H_0_ was variable up to H_0_ = 23,9 kAm^−1^. Measurements were made on 1 ml of sample at similar concentrations (of about 1.5 mg/mL), using a RF-immune temperature sensor to obtain the heating rates. To determine the SPA values of pure colloids (PAA-MNPs and LA-MNPs), the temperature increase of the samples were measured during 3–5 min, and the rate of temperature change extracted from the slope of the T vs. time curves.

### Cell culture

Immortalized BV2 cells from a murine microglial cell line (ATCC® CRL-2467^TM^) were cultured in Dulbecco’s modified Eagle’s medium supplemented with 10% fetal bovine serum, 100 IU/ml penicillin, 100 μg/ml streptomycin and 2 mM L-glutamine. Cells were maintained at 37 °C in a saturated humidity atmosphere containing 95% air and 5% CO_2_. The *in-vitro* experiments were designed at different concentrations of PAA-MNP and LA-MNPs. After the incubation time the cells were washed and the modified-DMEM was replaced with ordinary DMEM. Control experiments were performed with growth medium without nanoparticles.

### Cell Viability and Mnp Quantification Assays

For the cell viability experiments, 3 × 105 BV2 cells were seeded into a 6 well plate and incubated for 24 h at 37 °C with 5% CO_2_. The media was replaced with increasing magnetic nanoparticle concentrations of 0, 10, 20, 50 and 100 μg/mL for 24 hr. At the end of the incubation, the medium was removed and cells were washed three times with 2 ml of phosphate-buffered saline (PBS) then trypsinized, centrifuged and finally re-suspended in DMEM. Trypan blue assay was conducted by diluting 20 μl of cell samples into trypan blue (1:1). The viable cells were counted (unstained cells) differenced from those that were stained on blue (death cells).The % cell viability in respect to the control well was calculated whereby the control well was assumed have 100% viability. Quantification of uploaded PAA-MNP and LA-MNPs was analyzed from both the total amount and amount per cell of magnetic material incorporated. For those experiments, 106 cells were seeded in T25 flasks and incubated for 24 h. At that point the DMEM was replaced with DMEM containing 10, 20, 50 y 100 µg/ml of PAA and LA-MNPs. After 24 h of incubation the cells were washed with PBS, (3 × 2 ml) and then trypsinized. The pellet of the cells which was collected by centrifugation was then digested in HCl and HNO3. The amount of MNPs associated to the cells was quantified by UV-VIS spectrometry, using the same complexation reaction described above.

### Transmission Electron Microscopy and Dual Beam (Fib-Sem) Analysis

To prepare the samples for TEM analysis, an initial amount of 106 BV2 cells were seeded in T25 flasks. After 24 h of incubation with PAA-MNP and LA-MNPs (10 µgml−1) for 24 hours, the medium was removed and the cells were detached and fixed with 2% glutaraldehyde solution for 2 h at 4 °C. Then they were washed three times in cacodylate buffer (pH 7.2) and treated with potassium ferrocianate 2.5% and osmium tetraoxide 1% for 1 hour at room temperature. After washing, cells were dehydrated with increasing concentrations of acetone 30% (x2), 50% (x2), 70% (x2), 90% (x2) followed by further dehydration with acetone 100%. After drying samples were embedded in a solution (50:50) of EPOXI resin and acetone (100%) overnight, and then for 4–5 hours in resin EPOXI 100%. Sample were dried for 2 days at 60 °C and then cut in 70 nm thin slices. The samples were analyzed by transmission electron microscopy (TEM) using a FEI Tecnai T20 microscope and operating at 200 keV. STEM-HAADF images were obtained in a FEI Tecnai F30 microscope operated at an acceleration voltage of 300 kV. The microscope was equipped with a HAADF (high angle annular dark field) detector for STEM mode and EDX (X-ray energy disperse spectrometry).

The same protocol was used to prepare TEM and Dualbeam samples after MHT and WB application to BV2 cells and BV2 cells loaded with PAA-MNPs.

For FIB-SEM experiments, the samples were prepared by seeding 7,5 × 104 BV2 cells on a cover slip and incubating them 24 h with 10, 50 y 100 µg/ml of PAA-MNPs and 20 y 50 µg/ml of LA-MNPs. Then the samples were fixed with 2.5% glutaraldehyde in 0.1 M sodium cacodylate and 3%sucrose solution for 90 min at 4 °C. The dehydration process was carried out by incubating the cells for 5 min with increasing concentrations of methanol. The dual-beam FIB/SEM images were taken in a Nova 200 NanoLab (FEI Company), at 5 - 30 kV with a FEG column, simultaneously with a Ga-based 30 kV (10 pA) ion beam used to cross-sectioning single target cells. The analysis was completed by energy-dispersive x-ray spectroscopy (EDX) for specific determination of local chemical composition. Before observation, all samples were sputter-coated with a Pt layer of ≈10 nm thickness.

### Magnetic and Water-Bath Hyperthermia Experiments on Bv2 Cells

AMF and water-bath experiments were carried out on control BV2 cells and BV2 containing PAA-MNPs. 1 × 10^6^ cells were seeded for 1 overnight at 37 °C and 5% of CO_2_. The cells were incubated with PAA-MNPs at concentration of 100 μg/mL, and also a control sample of cells was grown under normal conditions for 24 h. Afterwards the cells were washed with PBS (3 × 2 ml) and enzymatically detached (Trypsinization). After centrifugation, the pellet of cells contained approximately 9 × 106 cells; it was resuspended in 150 μl of DME, and introduced in a 200-μl PCR tube. For the AMF experiments the PCR tubes were placed in the center of a thermally-insulated copper coil within the magnetic applicator device, and exposed to the AMF (f = 560 kHz and H= 23,9 kA/m) for 30 min at the constant target temperature T = 46 °C. The magnetic field applicator was capable to keep the target temperatures within ±0.5 °C by a feedback control of the magnetic field amplitude. Immediately after the field exposure and 4 hours after treatment, cell viability was measured using trypan blue. The trypan blue assay was conducted by diluting 20 μl of cell samples into 0.4% trypan solution (1:1). Each experiment was performed by triplicate. For the water bath experiments, the test tube containing the control BV2 cells and BV2 loaded with PAA-MNPs were placed in a hot water at constant temperature (46 °C) for a period of 30 min. Immediately and after 4 h of hyperthermia treatment the cells viability was quantified.

## Electronic supplementary material

Supplementary Information
