# Supplementary material for: Cell damage produced by magnetic fluid hyperthermia on microglial BV2 cells
Source: Sci Rep. 2017 Aug 17;7:8627. doi: 10.1038/s41598-017-09059-7 (PMC5561037; doi:10.1038/s41598-017-09059-7)
Supplement: Supplementary file 1 — Supplementary Information [file 41598_2017_9059_MOESM1_ESM.pdf]

Cell damage produced by magnetic fluid hyperthermia on microglial BV2 cells

M. Pilar Calatayud[1,2], Elisa Soler[1], Teobaldo E. Torres[1,3], Enrique Campos[1,], Concepción Junquera[4], M. Ricardo Ibarra[1,2,3], Gerardo F. Goya[1,2,†]*

1. Nanoscience Institute of Aragón, University of Zaragoza, Spain;
2. Department of Condensed Matter Physics, University of Zaragoza, Zaragoza, Spain.
3. Laboratory of Advanced Microscopies (LMA), Zaragoza, Spain.
4. Institute for Health Research Aragon IIS. Department of Biology. Faculty of Medicine. Zaragoza, Spain.

[*] Permanent address: Departamento de Física, CINVESTAV-IPN. Col. San Pedro Zacatenco, 07360 México City, México.

[†] Corresponding author: goya@unizar.es

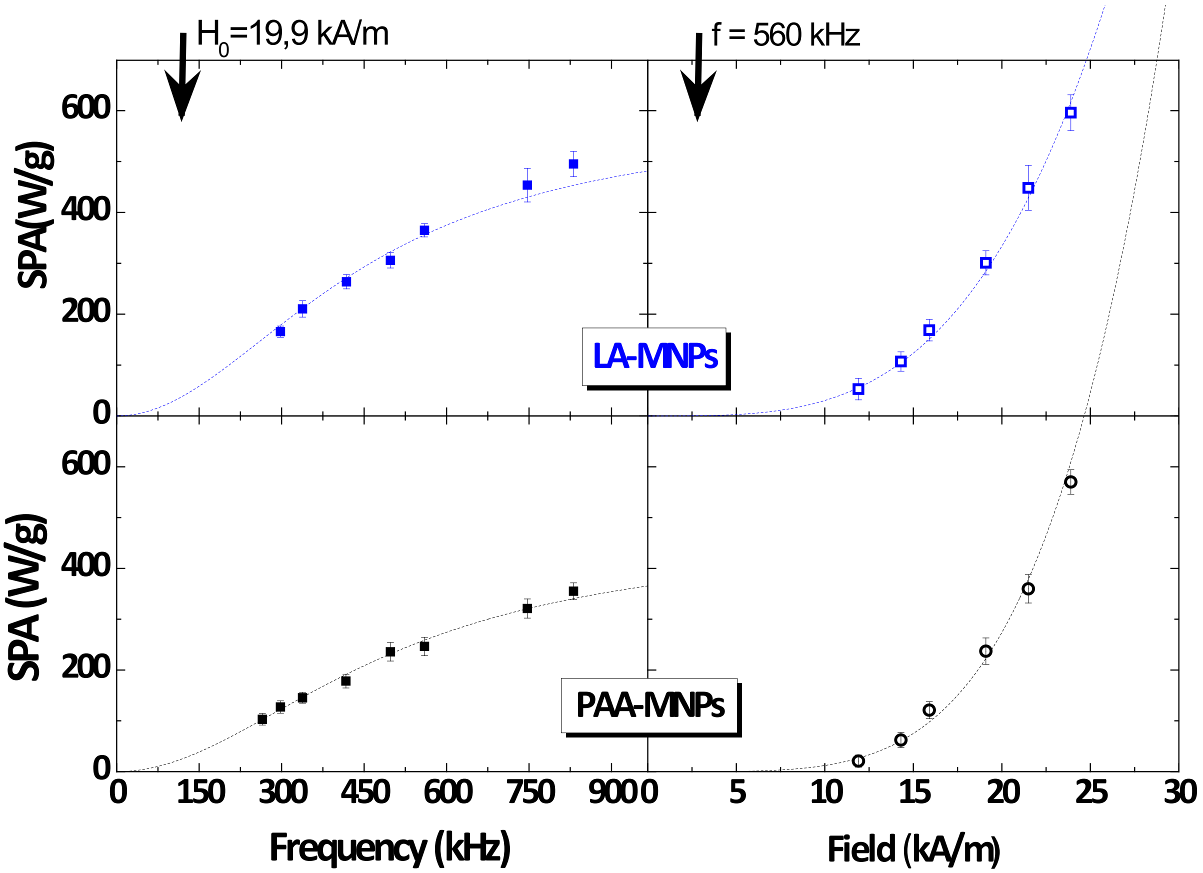

Figure S1. SPA measured (n = 3=) within the frequency range of 229-831kHz and amplitudes of the magnetic field of 3,9 -24,9 kA/m for both samples dispersed in water. The dashed lines are the fits of experimental data using the expression $\boldsymbol{SPA(H_0, f) = AH_0^2\left(\frac{Bf^2}{1+(Bf)^2}\right)}$ (see also equation 1 in the text).

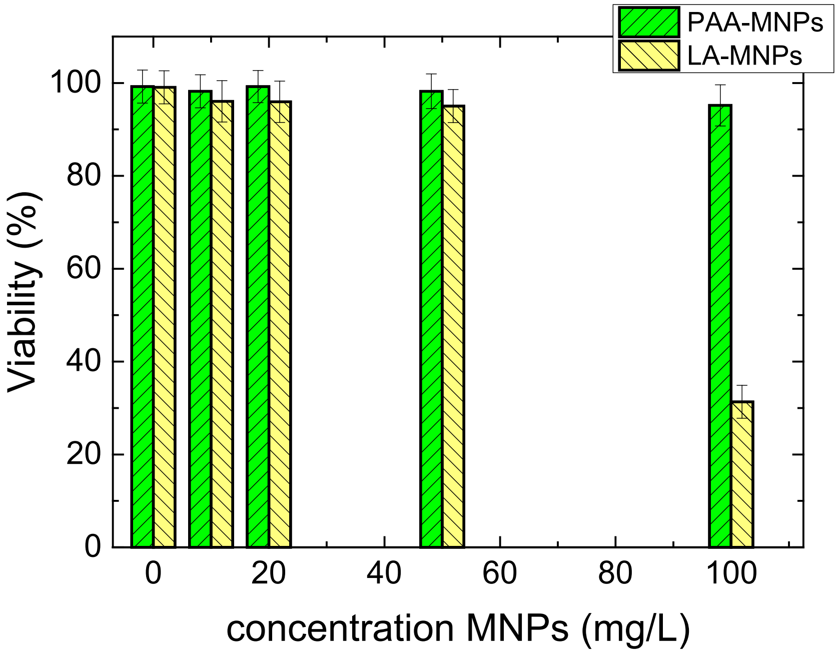

Figure S2. Viability of BV2 cells as measured by Trypan blue exclusion assay. The cells were treated with PAA-MNPs and LA-MNPs particles during 24 h in concentration values from 10 to 100 μg/mL.

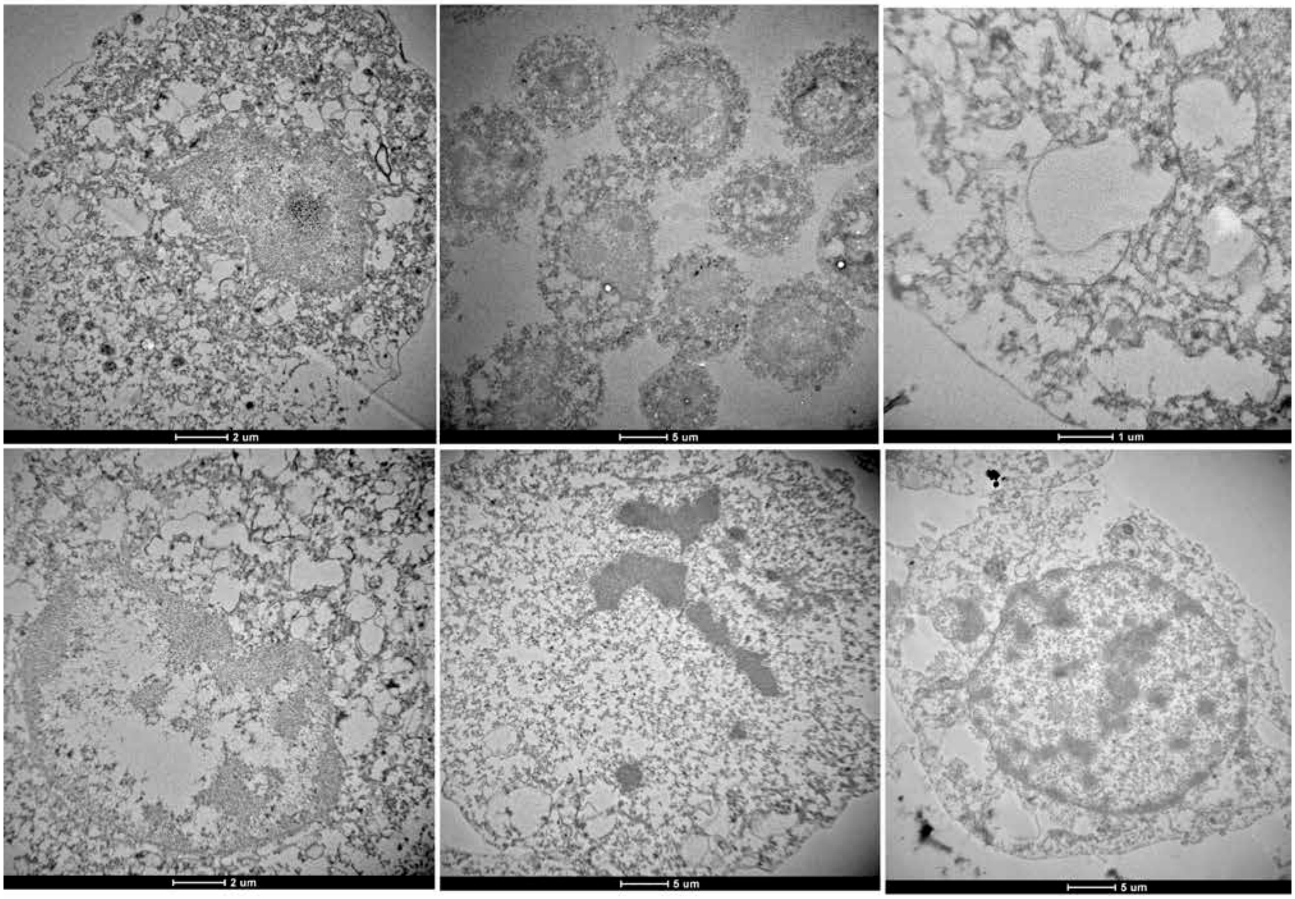

Figure S3. Transmission electron microscopy (TEM) images of BV2 cells 4 hours after WB heating during 30 min at 46ºC

.

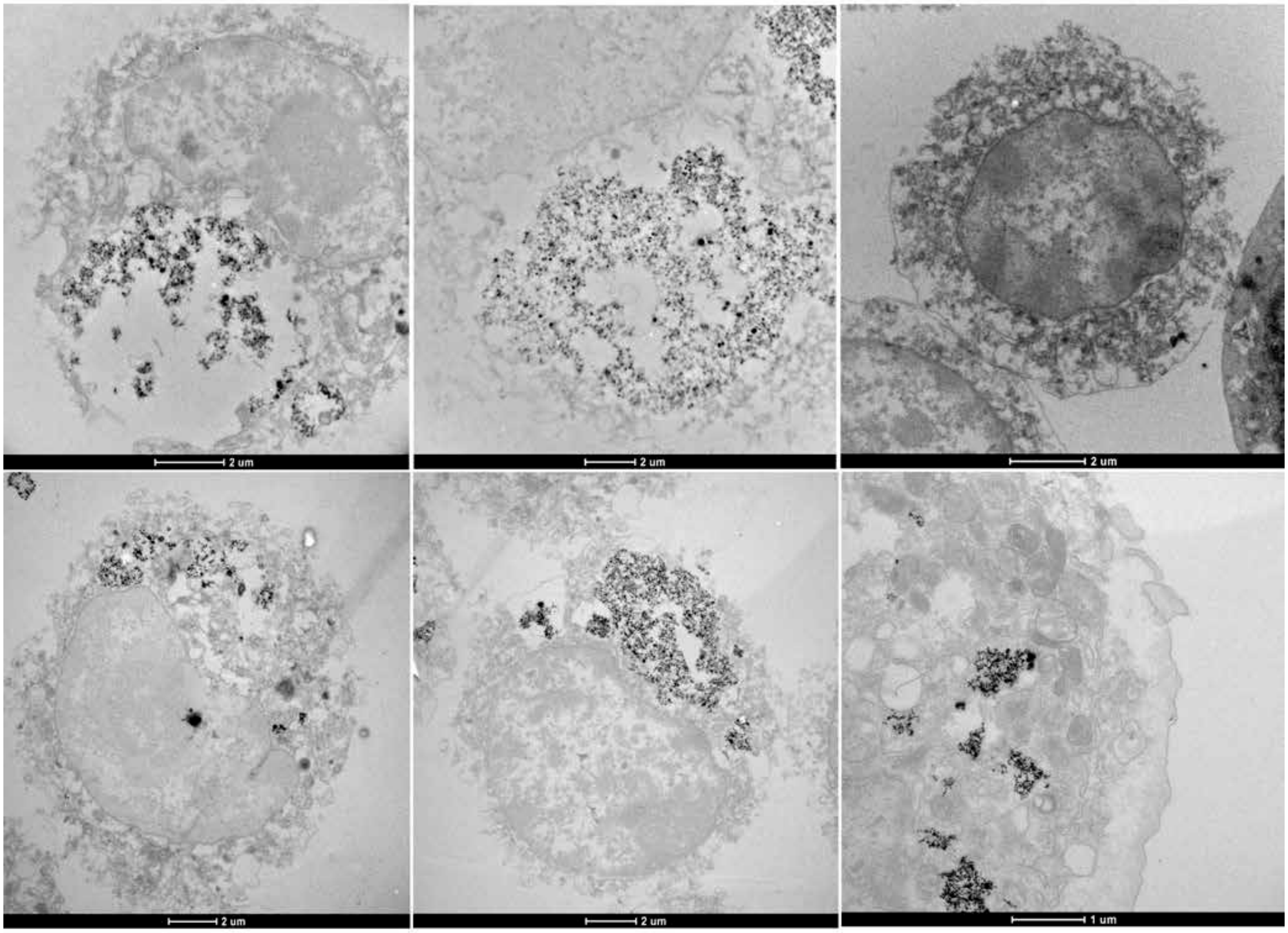

Figure S4. Transmission electron microscopy (TEM) images of BV2 cells incubated for 1 overnight with 100 µg/ml of PAA-MNPs and heated up to 46ºC during 30 min by WB.

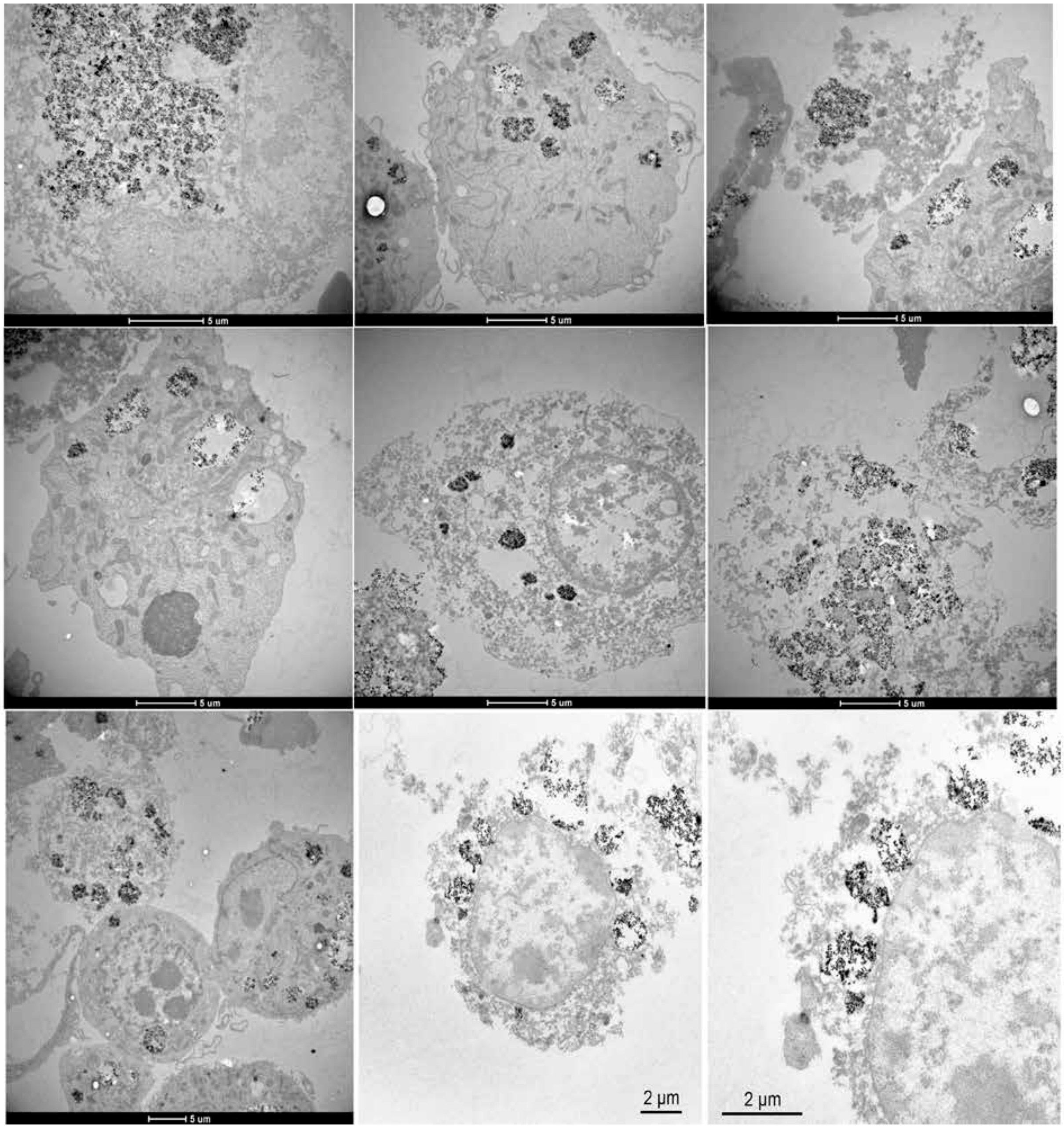

Figure S5. Transmission electron microscopy (TEM) images of BV2 cells incubated for 1 overnight with 100 µg/ml of PAA-MNPs and heated up to 46ºC during 30 min by AMF.
